# High-Resistant Packaging EPDM/SEBS Blends Processed by γ-Irradiation

**DOI:** 10.3390/foods15071151

**Published:** 2026-03-27

**Authors:** Traian Zaharescu, Ioana Cîrstea, Radu Mirea

**Affiliations:** 1 National Institute for Electrical Engineering, Radiochemistry Center (INCDIE ICPE CA), 313 Splaiul Unirii, 030138 Bucharest, Romania; ioanacirstea3@gmail.com; 2 Romanian Research and Development Institute for Gas Turbines—COMOTI, 220D Iuliu Maniu Blvd, 061126 Bucharest, Romania; radu.mirea@comoti.ro

**Keywords:** ethylene–propylene–diene monomer (EPDM), styrene–ethylene–butylene–styrene (SEBS), stability, γ-irradiation, chemiluminescence, gel content

## Abstract

The current paper aims to study the thermal stability of ethylene–propylene–diene monomer (EPDM) and styrene–ethylene–butylene–styrene (SEBS) block copolymer attained by radiation processing and evaluated by chemiluminescence (CL). Three blends with different weight ratios (1:3, 1:1 and 3:1), as well as individual rubbers, are γ-irradiated at 25, 50, 100 and 150 kGy. Their thermal stabilities are intercompared, and the activation energies required for oxidative degradation are calculated by using the values of oxidation induction times. Another investigation concerning the development of gel is in good agreement with the CL results. The aspects related to the mechanisms of the radiation fragmentation of blended components are discussed. The contributions of the blending components are evaluated based on the peculiar kinetic parameters, namely oxidation induction time (OIT) and onset oxidation temperatures (OOTs). It is clearly demonstrated that the EPDM component serves as the main source of radicals required for crosslinking, while the SEBS skeletons become the structural frames for the new crosslinked branches. The activation energies increase from 50 kJ mol^−1^ to 59 kJ mol^−1^ for unirradiated materials, but the increase for the blends exposed to 100 kGy is significantly larger from 41 kJ mol^−1^ to 54 KJ mol^−1^. The growth in the blending proportion of SEBS improves the thermal stability of the resulting materials. It is observed that the largest differences in the thermal resistances of γ-irradiated compounds are obtained for the samples exposed at 150 kGy, when the participation of each of the components is taken into account. This study highlights the research on and the productive methods of polymer processing, and the study of the irradiation of blends generates high-performance technical articles by the appropriate selection of technological parameters.

## 1. Introduction

Food-handling applications that require polymer materials that show enhanced mechanical strength are of considerable interest, where extending service life and limiting gas permeability are critical features of successful implementation [1,2]. These properties are governed by structural characteristics, whose modifications involve an alteration in the interfacial interactions with food products. For the manufacture of high-performance packaging materials, the radiation processing of polymers necessitates versatile technologies with significant potential for converting raw materials into well-defined products, such as capsules, sheets, bottles, and containers [3,4].

Accordingly, the evaluation of product stability is widely regarded as a fundamental requirement for the validation of radiation-based technologies [5,6]. The complexity of polymer processing under high-energy irradiation arises from the induced modifications of functional characteristics, which are possible in multicomponent material systems. As a promising approach, the radiation processing of packaging materials enables the production of high-performance products exhibiting enhanced chemical and biological resistances [7]. Moreover, this technology facilitates the assessment of ageing phenomena [8], an improvement in durability [9], and qualification for specific applications [10,11].

The interaction of ionizing radiation with polymer blends induces molecular chain scission, leading to degradation [12], or alternatively promotes crosslinking [13], the compatibilization of blend constituents [14], and composite formation [15]. The effective control over the generation and decay of free radicals is essential, particularly for the mitigation of oxidative processes that adversely affect long-term material performance [16]. Radiation processing must satisfy several technical requirements including a controlled increase in the gel fraction, the efficient compatibilization of blend components, and the suppression of oxidation through improved diffusion characteristics. An enhancement in mechanical properties can be achieved during the high-energy exposure of polymer systems, proving that the gelation dose remains within moderate limits. Concurrently, degradation via radical-mediated pathways may proceed at a higher rate than that of crosslinking [17] when oxygen-containing species accumulate, initiating the structural reorganization of the polymer substrate.

Polymer blends can yield materials with enhanced performance that are in line with the goals of radiation processing [12,18,19,20]. Accordingly, their constituent phases attain improved compatibility through interaction within homogeneous domains. During high-energy exposures, the controlled decay of irradiation-generated intermediates can be monitored by electron spin resonance (ESR) spectroscopy [21], chemiluminescence (CL) analysis [22], photoluminescence measurements [23], and differential scanning calorimetry (DSC) [24], whose corroboration with the resulting data is strongly influenced by radiochemical scission yields [25]. Molecular modifications in irradiated polymer substrates are initiated by the recombination of primary fragments progressing due to the reactivity and availability of transient intermediate species.

Numerous polymer systems containing polyolefin components have demonstrated substantial improvement upon high-energy irradiation [26]. Mechanistic interpretations based on free radical reactivity, as evaluated by electron spin resonance spectroscopy and chemiluminescence investigations, elucidate the followed radical decay pathways and account for the evolution of oxidative processes or the formation of extended crosslinked networks [27]. The development of high-performance materials is critically dependent on radical diffusion throughout the bulk material [28]. This ensures the effective compatibilization of blended phases.

Radiation processing provides multiple routes for the industrial valorization of polymeric materials [29] and for tailoring materials to exhibit specific functional properties [30]. The extension of this approach to diverse blend categories enables the attainment of application-oriented characteristics through various technologies in the automotive, aerospace, and rail transportation sectors; wire and cable insulation; heat-shrinkable tubing; biomedical devices; drug delivery systems; nuclear and space applications; and waste recycling. The feasibility of radiation-based manufacturing for polymer products intended for efficient packaging [31] is further supported by the widespread implementation of radiation sterilization for potentially contaminated food products [32].

**Figure 1:**
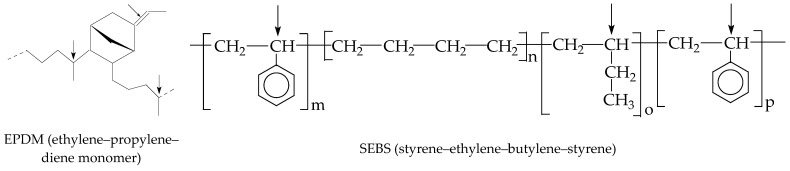
The molecular structures of blended polymers.

The primary objective of the present study is to investigate the effects induced by γ-irradiation on the stability–composition relationship of EPDM and SEBS rubber blends designed for food packaging applications, where γ-irradiation initiates interfacial vulcanization processes. The initial molecular structures, indicating radiation-sensitive sites, are presented in Figure 1.

The selection of these two polymers is closely associated with their relative radiation resistance. EPDM, a representative polyolefin elastomer, contains ethylene and propylene units with differing radiation stabilities, primarily due to the presence of tertiary carbon atoms that constitute the most vulnerable sites [33]. In contrast, SEBS exhibits greater radiation stability than the aforementioned elastomer [34]. The blends based on PP and EPDM display an enhanced tendency toward crosslinking under irradiation due to a high content of substituted tertiary carbon atoms [34]. Several SEBS-containing blends, such as PP/SEBS [35], PLA/SEBS [36], and LDPE/SEBS [37], demonstrate the available potential of radiochemical strategies to transform polymer blends into suitable food packaging materials, where both constituents interact via free radical mechanisms.

The energy input provided by γ-irradiation promotes the efficient compatibilization of the investigated components when oxidative degradation is retarded. The studied blends may represent relevant model systems for many other materials including polymeric wastes, whose recycling through radiation processing could contribute to several circular economy strategies. The present findings may also constitute significant reference points for the fabrication of composite materials, as indicated in previous reports [38]. The extension of this methodology for composite processing [39] opens new perspectives for the valorization of polymer blends in nuclear applications, such as radiation-shielding materials. The availability of reactive sites suitable for crosslinking was previously demonstrated [33], supporting the applicability of radiation processing across various polymer classes within a broad irradiation dose range (20–100 kGy). The structural features of both blend components facilitate crosslinking reactions, while the evolution of curing and enhanced radiation resistance is evidenced by gel fraction development [33] correlated with the high proportion of substituted tertiary carbon atoms that confer resistance to oxidative degradation.

The subject of this paper is an example of appropriate technology for the manufacture of various packaging materials. Based on technical reproducibility, the large variety of processed structures, and the facile conversion of polymer blends into versatile composites, the materials modified by γ-irradiation show good performance, including low diffusion rates of gases and extended durabilities. The polymer pairs consisting of a polyolefin and an elastomer solve the problems of processability, which involves the thermal range of the manufacture of products, the vast applications related to environmental conditions, and the implementation of this procedure for other compositions that generate useful items, especially in medicine. The study of the compatibilization of blends consisting of EPDM and SEBS allows for the identification of several applications, except packaging materials, for example, the anticorrosive applications of metallic surfaces and the production of electrical insulation, soundproofing materials, flexible belts, and domestic goods. However, the main goal of this study is the demonstration of the basic role of the blending composition that determines the stability features of the final product.

## 2. Materials and Methods

### 2.1. Materials

The raw polymers, ethylene–propylene–diene monomer (EPDM) and styrene–ethylene–butylene–styrene (SEBS) block copolymer, were provided by ARPECHIM Pitești (Romania) and Kuraray Europe France S.A.R.L., Bourg-La-Reine (France), respectively. While the styrene/rubber ratio in SEBS is 35/65, the ethylene/propylene ratio in EPDM is 70/30. The fraction (3.5 wt%) of 2-ethylidene-5-norbornene is present in EPDM as the diene content.

### 2.2. Sample Preparation

The samples were prepared by the dissolution of solid polymers in CHCl_3_ (chloroform) produced by Reactivul (Bucharest, Romania) as a pro analysi reagent in separate glass bottles for each concentration. After the disposal of solid polymer components in the intended ratios, 50 mL of solvent was added in the preparation bottles. These flasks were vigorously shaken until homogenous solutions were obtained. Then, small volumes (20 mL) were transferred in aluminum trays (diameter 7 mm) by a micropipette, and smooth solvent evaporation for 2 h at room temperature was accomplished gently. Thus, thin films of about 2–3 mg were obtained in a dry state and with a thickness of 50 μm. This thickness does not contribute to the self-absorption of emitted CL photons. Five types of compositions were prepared: neat polymers (EPDM and SEBS) and three EPDM/SEBS mixtures (3/1, 1/1 and 1/3 wt%). This blending range offers a general view on the contribution of each component.

### 2.3. γ-Irradiation

γ-exposure was achieved in an irradiation machine (Ob Servo Sanguis, Budapest, Hungary) provided with a ^60^Co source, whose dose rate is 0.5 kGy h^−1^. The exposures were accomplished in air at room temperature. Four γ-processing doses (0, 25, 50, 100, and 150 kGy) were applied for the characterization of the thermal and radiation resistances of the studied specimens.

### 2.4. Methods

#### 2.4.1. Chemiluminescence Assay

Chemiluminescence (CL) measurements as nonisothermal determinations at four heating rates, 5, 10, 15, and 20 °C min^−1^, and an isothermal assay conducted at 160 °C, 170 °C and 180 °C were performed using the LUMIPOL 3 unit produced by the Institute of Polymers, Slovak Academy of Sciences, Bratislava, Slovakia. While the OOT from nonisothermal determinations characterizes the temperature, where a measurable increase in CL intensity appears, the OIT is the time when oxidation starts in isothermal measurements. Kinetic parameters are obtained by the intersection of the Ox axis with the tangent drawn at the curve on the propagation stage. All CL measurements were carried out in air in static conditions. For the CL measurements, only one replicate was recorded, because the measurement error is very low (±0.1%), and data reproducibility is quite high. CL determinations were done immediately after the end of irradiation. CL spectra were recorded for the characterization of structural variation by the modification of the exposure irradiation dose. Compositional parameters determine the shapes of evolution for various oxidated states, when the temperature applied in the heating zone increases and the degradation of organic substrate is accelerated starting from the levels reached after γ-irradiation. The minimal correlation factor is 0.98687, as revealed by the calculation of linearity, which demonstrates the accuracy of experimental measurements. This factor is the result of CL measurements and the determination of OIT from the dependence of CL intensity on ageing time. This error is very low, because measurement accuracy is in the range ±0.25%. Additionally, the measurement errors are at this level because the device used allows for the fine adjustment of tension for the photon detection of the photomultiplier. The values of the required activation energies were calculated by means of the Arrhenius relationship Equation (1) starting from the values of oxygen induction times obtained from the isothermal chemiluminescence measurements on polymer samples,

(1) $$\ln k=\ln A-\frac{E_{a}}{RT}$$

where *k* and *A* are the rate constant and the frequency factor of oxidative degradation, respectively; *E_a_*, *R* and *T* are the values of activation energy, the universal constant of gases and the testing temperature, respectively. By the representation of the logarithmic values of oxygen induction times versus the reciprocal values of absolute testing temperature, the values of activation energies were calculated from the ratio *E_a_*/*R*, the slope of the obtained line.

#### 2.4.2. Evaluation of Gel Content

Gel content determinations were achieved by boiling dense copper fabric bags containing a pre-weighted sample (*m*_0_), with an initial sample weight of about 0.1 g, in *o*-xylene in a glass Soxhlet extraction unit for 24 h, where a solvent volume of 250 mL was boiled. Three replicates of each sample were analyzed. After washing and drying the sacs, new weights were obtained (*m_f_*). The percentage value of the gel fraction (*m_g_*) was calculated by the following relationship (2):

(2) $$m_{g}=\frac{m_{f}}{m_{0}}\cdot 100$$

During this operation, 0.5 g of IRGANOX 1010 with the role of an oxidation inhibitor was added into the boiling *o*-xylene (250 mL). The Charlesby–Pinner representation, denoting the dependence of the soluble fraction on the reciprocal dose, was drawn after the calculation of these amounts by Equation (3):

*S* = 1 − *P* = 1 − *m_g_*/100 (3)

where *S* represents the parts of the soluble fraction, and *P* is the gel fraction.

The error of gel measurements is ±0.5%, which represents the cumulation errors generated by two weighings and the applied drying.

## 3. Results

This study evaluates the homogeneous interpenetration of EPDM and SEBS as a strategy to obtain blends with enhanced oxidative stability, thereby mitigating accelerated ageing during and after γ-irradiation. The proposed system also represents a model for the radiation processing of polymer wastes, where synergistic coupling effects are advantageous: EPDM functions as a primary radical source, while SEBS provides a macromolecular framework capable of stabilizing and incorporating the generated radicals. The differences shown in the intrinsic radiation stability of the two components enable controlled structural development, in which radiolysis-derived fragments determine the network architecture as a function of blend composition.

Upon irradiation, free radicals are generated through the chain scission of propylene units and residual double bonds in EPDM [40], as well as butylene segments in SEBS [41]. These simultaneously formed reactive intermediates participate in concurrent oxidation and crosslinking reactions similar to the mechanisms reported for PP/HDPE blends containing SEBS [42]. The superior oxidation resistance of EPDM and SEBS is further supported by observations in crosslinked polyethylene (XLPE), where increasing the SEBS content reduces the formation rate of water trees [43]. The concurrent radical population within both blend phases promotes interfacial crosslinking, while the recombination reactions limit the accumulation of oxidized species. Moreover, the mediating function of SEBS in related systems, such as PS/HDPE blends, was previously demonstrated [43], highlighting its role as an effective structural support for crosslinking processes.

### 3.1. Evaluation of Gel Content

**Figure 2:**
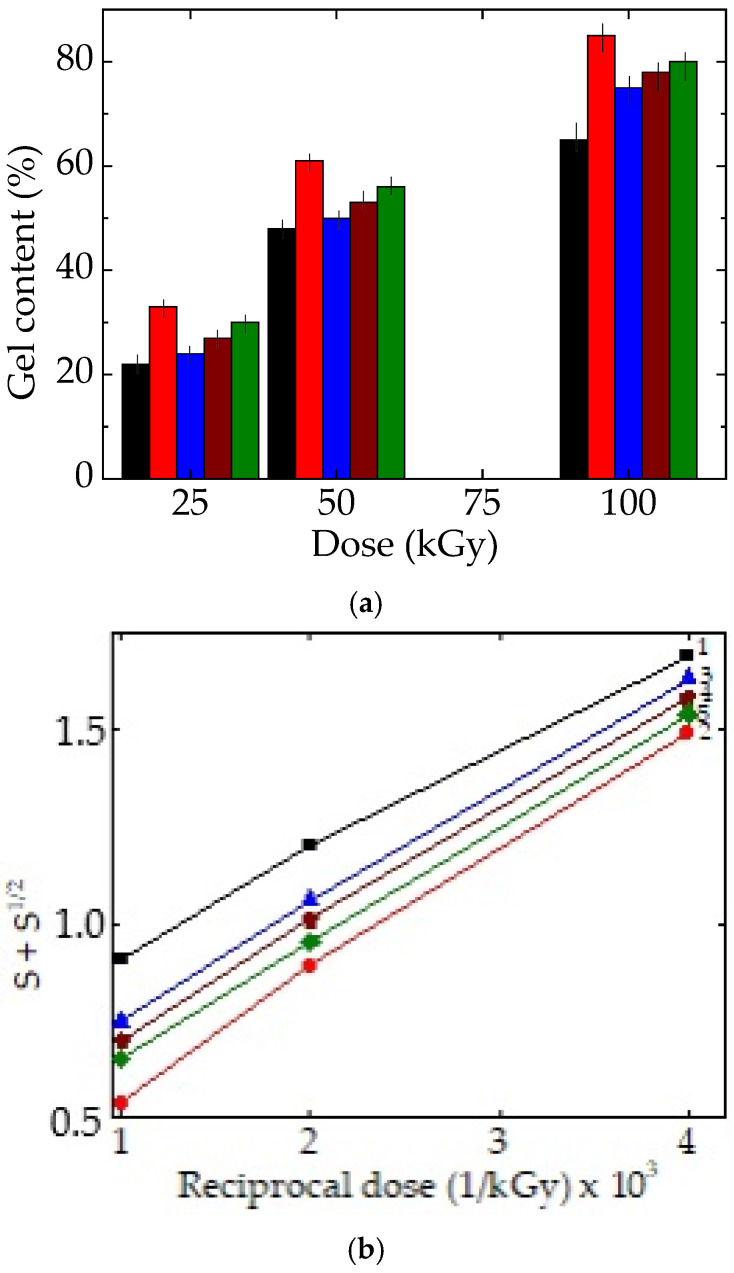
The gel content (**a**) and Charlesby–Pinner representation of the accumulation of the insoluble fraction (**b**). SEBS/EPDM ratio: (1, black) 0/100; (2, red) 100/0; (3, blue) 25/75; (4, wine) 50/50; (5, olive) 75/25.

During the exposure of polymers to ionizing radiations like γ-rays, the evolution of gel content depends on the ratio between the rates of scission and crosslinking. SEBS and EPDM belong to the crosslinking class of polymers [44]. The ascendent progress of the cured fraction is proof of the stability increase in the studied systems (Figure 2). This accumulation is controlled by the values of component contents. The increase in the proportion of SEBS determines the faster development of gel content, which hinders the diffusion of oxygen involved in oxidative degradation.

The differences that exist between the slopes of the obtained lines describe the increase in the crosslinking proportion in SEBS with respect to EPDM. This means that the ethylene–propylene elastomer plays the role of the main radical source that is jointed on the backbones of SEBS. The correlation between oxidative degradation and crosslinking makes the γ-irradiated materials turn towards the stabilized zone and, at the same time, indicates that radiation exposure is a suitable technology for the processing of food packaging polymer materials. Irradiation doses exceeding 100 kGy initiate advanced ageing due to the increase in the concentration of radicals, which would produce advanced oxidation.

γ-exposure induces the amelioration of gas penetration into the whole material volume, and it is also an excellent procedure for the diminution of bacteriological contamination [45]. This treatment does not affect the nutritive qualities of food, because the dose is low (less than 25 kGy) and the oxidation products are retained in the polymer matrices [46]. Good food preservation conditions are achieved by γ-irradiation due to the mitigation of degradation consequences under unproper storage and the extension of shelf life.

### 3.2. Chemiluminescence Determinations

**Figure 3:**
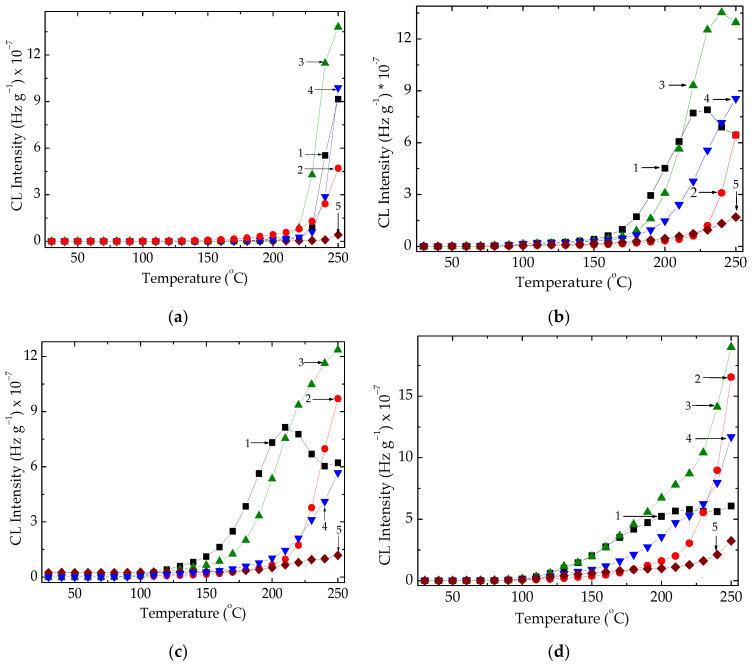
Nonisothermal chemiluminescence (CL) spectra of EPDM/SEBS samples recorded at a heating rate of 10 °C min^−1^. Formulations for EPDM/SEBS samples: (1) 100/0; (2) 25/75; (3) 50/50; (4) 75/25; (5) 0/100. Irradiation at (**a**) 0 kGy, (**b**) 50 kGy, (**c**) 100 kGy, and (**d**) 150 kGy.

The chemiluminescence studies conducted provide information on the progress of oxidation, which characterizes the contributions of polymer phases to attaining a certain degradation state [47]. For the evaluation of thermal stability, the nonisothermal determinations disclose not only the values of onset oxidation temperature, the peculiar heating level, and when oxidation becomes measurable but also the trend in degradation as the material accumulates certain amounts of its oxidation products (Figure 3).

Figure 3 shows that the SEBS component exhibits the highest stability, while the EPDM fraction provides the largest number of free radicals. In the unirradiated materials (Figure 3a), the temperatures depicting the start of oxidation do not differ from one formulation to another, except OOT for pristine SEBS. Their degradation becomes significant when the temperature exceeds 212 °C. Moreover, the ethylene–propylene elastomer is damaged more easily than SEBS. When they are blended, the component proportion makes a difference in the progress of oxidation. The higher the SEBS content, the higher the resistance. Radiation exposure affects the compositions that include EPDM to a larger extent, because its unsaturation becomes the main reason for the scission of its backbones. During the radiolysis of the studied compositions, the generation of free radicals from the EPDM phase feeds the process of recombination in the SEBS fraction, increasing the thermal and radiation stabilities of the processed materials. Reliable proofs sustaining this statement are represented by the OOT values (Table 1), whose figures found for the increasing mixture percentage of SEBS are enhanced.

Simultaneously, the early progress of oxidation happens smoothly, indicating that the radical recombination is more probable than the oxidation process. The stability coexistence of the studied polymer pair demonstrates the efficiency of thermal and radiation processing for polymers, when one component has a higher scission yield than the second component. This complete behaviour is the basic direction in which the compatibilization of the studied components is achieved, when the components are suitable for efficient blending [28,48] and extended durability is obtained [49].

**Figure 4:**
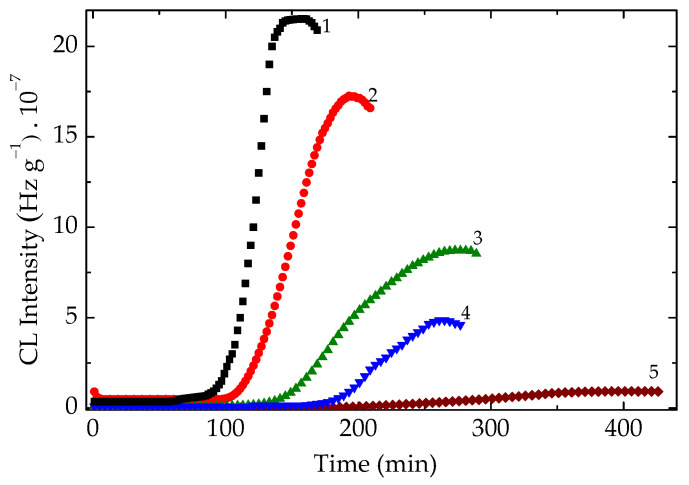
The isothermal CL spectra recorded on the unirradiated samples. Temperature: 160 °C. EPDM/SEBS proportions: (1) 100/0; (2) 25/75; (3) 50/50; (4) 75/25; (5) 0/100.

The isothermal chemiluminescence investigation allows us to identify the potential of radiation processing for the radiochemical modification in polymer blends, namely EPDM/SEBS. The values of oxidation induction times reflect the stabilization trend (Figure 4), while the values of activation energy suggest the contribution of the stable component to the increase in oxidation strength (Table 2), which supports the propagation of recombination.

The variation in OIT characteristics shown in Figure 4 may be interpreted as the capability of the intermediates from the EPDM phase to join on the SEBS backbones, which contain benzene nuclei, a screening factor during oxidation. The engineering applications of thermal testing on the structuration of polymer blends concern the amelioration of thermal resistance, when the scavenging activity of a more stable component is the mechanistic support. The inclusion of an efficient antioxidant or reactive filler becomes a supplementary stimulation for an advanced improvement in the thermal properties of the resulting materials, as this occurs in many other blends [50]. Taking into account the efficiency of the recombination process, the durability of the resulting materials is confirmed by the diffusion of radicals from one phase into another part. This behaviour is supported by the higher values of activation energy after the appropriate treatment.

The assistance of chemiluminescence as a reliable methodology represents an accurate, key assay, because it truly reflects the evolution of stability based on the particularly followed methods of decay for radiolysis intermediates. This type of stability applied to polymers may also be considered a proper tool for the characterization of functional performance.

## 4. Discussion

The compatibilization of EPDM and SEBS polymers is primarily achieved through radical-mediated interactions during γ-irradiation, coupled with a decrease in oxygen penetration due to the growth of an insoluble crosslinked fraction. The dynamic balance between crosslinking and oxidation is strongly influenced by the rate of radiolytic fragmentation, which governs the availability of reactive radicals within the system. The progressive increase in gel content, as illustrated in Figure 2a, and the concomitant elevation in the onset oxidation temperature (OOT) values reported in Table 1 highlight the crucial role of SEBS as the more thermal and radiochemical stable component within the blend. These observations indicate that the structural integrity and oxidative resistance of the material improve with a higher SEBS content and increasing irradiation doses.

Mechanistically, the enhancement in resistance in the EPDM/SEBS blends can be conceptualized using a recombination-based model, such as the Charlesby–Pinner representation shown in Figure 2b. The development of a larger insoluble fraction corresponds to a higher proportion of crosslinked material, which is reflected in the slight differences in the slopes of the gel growth curves. This mechanistic behaviour is further correlated with the presence of aromatic moieties, such as benzene rings in SEBS, which can absorb and store radiant energy, thus stabilizing the polymer backbone and facilitating crosslink formation. As previously reported in some studies of crosslinking mechanisms [33], the growth of the insoluble fraction is inversely related to the oxidation state of the system; free radicals are preferentially consumed in crosslinking reactions rather than in oxidative pathways. Consequently, the degree of oxidation decreases with an increasing SEBS content. This remark is consistent with analogous polymer systems, such as PE/SBS blends, where electron spin resonance (ESR) studies have shown that the peroxide content diminishes as the SBS phase fraction increases [51]. Therefore, a higher SEBS content directly contributes to lower oxidation levels, which is a critical consideration for the production of safe and long-life food packaging materials.

Radiation-based technologies offer a significant advantage for the manufacture of high-performance polymer materials intended for food packaging due to their ability to promote intimate blending and high degradation resistance. The primary benefit of radiation processing is an enhancement in the crosslinking fraction, which reduces the effective diffusion coefficients of oxygen and other gases within the polymer matrix. This reduction in molecular mobility slows down degradation rates, thereby extending the lifetime of packaging materials [52]. Additionally, γ-irradiation provides an effective sterilization mechanism, substantially lowering the risk of biological contamination in food packaging [32].

Importantly, radiation treatments generate only minimal quantities of oxidation precursors, such as peroxyl radicals, which do not significantly compromise the service life of products. At low irradiation doses, typically below 25 kGy, which correspond to standard sterilization requirements, the mechanical properties of packaging materials remain largely unaffected, ensuring that both the polymer and the contained food preserve their initial characteristics over prolonged periods [6]. Beyond mechanical stabilization, the application of radiation technologies also induces structural improvements, including enhanced crosslinking, increased surface hydrophilicity, and bio-protective effects. These modifications collectively reduce the mobility of residual degradation products and trace compounds, further contributing to the long-term stability and safety of polymer packaging [53].

Overall, the combination of radical-mediated compatibilization, crosslinking, and controlled oxidation offers a scientifically robust strategy for producing durable, high-performance EPDM/SEBS blends. The integration of these mechanisms through radiation processing not only enhances thermal and oxidative resistance but also provides additional functional benefits, including sterilization, gas barrier improvement, and the preservation of mechanical integrity, making such blends highly suitable for advanced food packaging applications.

## 5. Conclusions and Perspectives

The present investigation is a versatility demonstration of the radiation processing of polymer blends when a suitable stabilization of material is reached without attaining a certain disturbing level of oxidation. The analyzed methodology may become a general procedure for the manufacture of many categories of flexible products that address high oxidation resistance and increase reliability. Some identified features must be emphasized for the extension of the described findings:

Radiation processing represents an effective method for enhancing the thermal resistance of EPDM/SEBS blends, yielding materials with improved stability under oxidative conditions.

The two components exhibit complementary roles: EPDM acts as the principal radical source, while SEBS provides macromolecular backbones onto which the generated radicals recombine, promoting structural stabilization.

The kinetic parameters derived from chemiluminescence measurements under nonisothermal (onset oxidation temperature) and isothermal (oxidation induction time) conditions demonstrate a progressive improvement in thermal stability as the SEBS content increases from 25 wt% to 75 wt%.

An irradiation dose of 100 kGy represents a practical stability threshold for these systems; beyond this level, oxidation proceeds more gradually in blends containing higher SEBS fractions.

Increasing temperature accelerates degradation, when the oxidative decay of free radicals predominates over crosslinking reactions.

An energetic analysis of radiation effects on material durability indicates that the EPDM/SEBS 1:3 composition exhibits the highest OIT values, even at 100 kGy.

The coexistence of EPDM and SEBS in γ-irradiated blends enhances both thermal and radiation stability across all blending ratios, as confirmed by the activation energies determined from isothermal chemiluminescence measurements.

Gel fraction development and isothermal chemiluminescence analyses highlight the decisive contribution of SEBS to oxidative resistance, while EPDM ensures sufficient radical generation to mitigate ageing through controlled crosslinking.

The optimized composition is suitable for manufacturing long-life products and offers potential for polymer waste recycling through radiation processing.

The extension of radiation processing to other polymer blends with sufficiently different intrinsic radiochemical stabilities may enable the design of materials with tailored durability and performance. The present findings are particularly relevant for the production of flexible components, such as O-rings, buffers, and gaskets, intended for demanding environments including fluid circuits in nuclear power plants. Furthermore, EPDM- and SEBS-based systems may be adapted for applications requiring operation under harsh irradiation and thermal conditions.

These results may also stimulate further research into high-performance blends and composites with enhanced oxidative and radiation stability including advanced materials suitable for long-term service and specialized applications such as durable food packaging. This study itself is an availability demonstration of radiation processing, which may be applied to a large category of polymers.

## Figures and Tables

**Table 1:** The onset oxidation temperatures measured during the nonisothermal CL measurements of EPDM/SEBS specimens.

EPDM/SEBS	OOT (°C)			
	0 kGy	50 kGy	100 kGy	150 kGy
100/0	221	188	162	145
0/100	235	225	211	208
75/25	202	165	148	140
50/50	212	178	154	162
25/75	225	192	181	188

**Table 2:** The activation energies calculated from the results of isothermal chemiluminescence.

Dose (kGy)	Sample Formulation (EPDM/SEBS)	Oxidation Induction Time (min)			Correlation Factor	Ea (kJ mol^−1^)
		160 °C	170 °C	180 °C		
0	100/0	104	82	56	0.98932	50
	0/100	175	130	88	0.99578	56
	75/25	131	96	68	0.99908	53
	50/50	156	105	77	0.99838	58
	25/75	196	135	95	0.99999	59
100	100/0	61	45	37	0.99378	41
	0/100	72	50	42	0.98237	44
	75/25	61	43	35	0.99083	45
	50/50	71	52	39	0.99995	49
	25/75	82	57	42	0.99935	54

## Data Availability

The original contributions presented in this study are included in the article. Further inquiries can be directed to the corresponding author.

## Abbreviations

The following abbreviations are used in this manuscript:

*A*	Frequency factor of oxidative degradation
CL	Chemiluminescence
DSC	Differential scanning calorimetry
*E_a_*	Activation energy
EPDM	Ethylene–propylene–diene monomer
ESR	Electron resonance spectroscopy
FTIR	Fourier transform infrared spectroscopy
HDPE	High-density polyethylene
K	Rate constant of oxidative degradation
LDPE	Low-density polyethylene
*m_f_*	Final polymer mass of sample
*m_g_*	Percentage gel fraction
*m_o_*	Initial polymer mass of sample
OIT	Oxidation induction time
OOT	Onset oxidation temperature
*P*	Insoluble fraction
PLA	Poly(lactic acid)
PP	Polypropylene
PS	Polystyrene
R	Gas constant
*S*	Soluble fraction
SEBS	Styrene–ethylene–butylene–styrene copolymer
XLPE	Crosslinked polyethylene
